# Supplementary material for: Analysis of PPARγ Signaling Activity in Psoriasis
Source: Int J Mol Sci. 2021 Aug 10;22(16):8603. doi: 10.3390/ijms22168603 (PMC8395241; doi:10.3390/ijms22168603)
Supplement: Supplementary file 1 [file ijms-22-08603-s001.zip › Supplemental materials_Analysis of PPARg signaling activity in psoriasis/Pathway models/Models images and html files/Model of PPARG signaling in psoriasis/1007183897.html]

CXCL13 ---- CXCR5


# Binding CXCL13 ---- CXCR5

|  |  |
| --- | --- |
| URN | urn:agi-binding:in-out:urn:agi-llid:10563:in-out:urn:agi-llid:643 |
| Connectivity | 2 |
| References | 10 |

---
